# On the Use of Variance per Genotype as a Tool to Identify Quantitative Trait Interaction Effects: A Report from the Women's Genome Health Study

**DOI:** 10.1371/journal.pgen.1000981

**Published:** 2010-06-17

**Authors:** Guillaume Paré, Nancy R. Cook, Paul M. Ridker, Daniel I. Chasman

**Affiliations:** 1 Department of Pathology and Molecular Medicine, Population Genomics Program, McMaster University, Hamilton, Canada; 2 Department of Preventive Medicine, Brigham and Women's Hospital, Boston, Massachusetts, United States of America; Princeton University, United States of America

## Abstract

Testing for genetic effects on mean values of a quantitative trait has been a very successful strategy. However, most studies to date have not explored genetic effects on the variance of quantitative traits as a relevant consequence of genetic variation. In this report, we demonstrate that, under plausible scenarios of genetic interaction, the variance of a quantitative trait is expected to differ among the three possible genotypes of a biallelic SNP. Leveraging this observation with Levene's test of equality of variance, we propose a novel method to prioritize SNPs for subsequent gene–gene and gene–environment testing. This method has the advantageous characteristic that the interacting covariate need not be known or measured for a SNP to be prioritized. Using simulations, we show that this method has increased power over exhaustive search under certain conditions. We further investigate the utility of variance per genotype by examining data from the Women's Genome Health Study. Using this dataset, we identify new interactions between the *LEPR* SNP rs12753193 and body mass index in the prediction of C-reactive protein levels, between the *ICAM1* SNP rs1799969 and smoking in the prediction of soluble ICAM-1 levels, and between the *PNPLA3* SNP rs738409 and body mass index in the prediction of soluble ICAM-1 levels. These results demonstrate the utility of our approach and provide novel genetic insight into the relationship among obesity, smoking, and inflammation.

## Author Summary

Finding gene–gene and gene–environment interactions is a major challenge in genetics. In this report, we propose a novel method to help detect these interactions. This method works by first identifying a subset of genetic variants more likely to be involved in genetic interactions and then testing these variants for interaction effects. Using this method, we were able to identify three previously unknown genetic interactions. The first interaction involves a measure of body fat and a genetic variant of the *LEPR* gene in the prediction of C-reactive protein concentration, a marker of inflammation. The second interaction involves the same measure of body fat and a genetic variant of the *PNPLA3* gene in the prediction of ICAM-1 levels, also a marker of inflammation. These results are significant because both *LEPR* and *PNPLA3* are linked to the biological response to increased body fat, and inflammation itself is known to be increased in obesity and is thought to contribute to its adverse health effect. Finally, a third interaction was identified between a genetic variant of the *ICAM1* gene and smoking in the prediction of ICAM-1 levels. The *ICAM1* gene encodes ICAM-1 itself and smoking is known to be an important determinant of ICAM-1 concentrations.

## Introduction

Most genetic association studies have so far emphasized the effect of SNPs on mean values of a trait of interest. While this approach has been widely successful in identifying genetic determinants of quantitative traits, it does not make use of differences in the variance of these traits per genotype, which may reflect an alternative mode of genetic influence and signify biological relevance. Several scenarios can result in the variance of a quantitative trait being different when individuals are stratified by SNP genotype (or any other genetic variation) in cross-sectional designs. For examples, such difference could be ascribed to increased variation over time among individuals of a given genotype, increased variation in the homeostatic set-point of a trait (without increased variation over time), or an interaction effect with another SNP or exposure. This latter possibility is particularly attractive since information on variance per genotype could thus be leveraged to reduce the dimensionality of interaction analysis by prioritizing SNPs for further interaction testing.

Several methods have been developed to address the statistical challenge of assessing the degree and nature of interaction between two or more genetic loci [1]–[4]. While these offer invaluable insight, most do not address the main problem pertaining to interaction testing in the context of whole genome association studies: the multiple hypothesis testing incurred when searching for interactions between hundreds of thousands of genetic variants. In theory, excluding unlikely interactions on an *a priori* basis will result in a lower number of false positives tests and increased power. Current data reduction methods use evidence of co-adaptation between loci to restrict the search space [5] or specify a minimum marginal effect to select a subset of SNPs for interaction testing [6]–[8]. This latter method is based on the assumption that interacting SNPs will show at least a moderate effect on the trait of interest. While this premise might be valid in most cases, it need not be, as has been observed [2], [9], [10]. Furthermore, even when restricting the search for interaction to SNPs with marginal effect P-values lower than 0.1, as has been suggested [7], a sizeable number of hypotheses will have to be tested. Thus there is a need for novel methods to identify SNPs likely to be involved in gene-gene and gene-environment interactions. Murcray et al. [11] recently proposed a two-step approach to test for gene-environment interactions in genome-wide association studies. In the context of dichotomous outcomes, this method first tests for gene-environment association, followed by conventional interaction testing of the subset of markers with significant association in the combined case-control sample.

In this report, we first derive expressions for the variance according to SNP genotype under models of genetic interactions and demonstrate that the variance of a quantitative trait will differ between the three possible genotypes of a biallelic SNP in the presence of genetic interactions. Building on these derivations, we then simulate data to explore contexts where testing for inequality of variance between genotypes could be used to prioritize SNPs for further interaction testing. Leveraging these observations, we propose a novel method to prioritize SNPs for gene-gene and gene-environment interaction, “variance prioritization”. Variance prioritization entails two steps. In the first step, Levene's test of equality of variance is used to prioritize SNPs for further interaction testing. In the second step, prioritized SNPs are tested for interaction effects against environmental covariates or other SNPs using linear regression. We use data from the Women's Genome Health Study (WGHS) on two inflammation markers, C-reactive protein and soluble ICAM-1, to test whether (1) there is empirical confirmation of SNPs demonstrating inequality of variance between genotypes and (2) whether such SNPs show evidence of either gene-environment or gene-gene interactions.

## Methods

### Ethics statement

All analyses were performed with approval of the institutional review board of the Brigham and Women's Hospital. All members of the WGHS cohort were participants in the WHS who provided an adequate baseline blood sample for plasma and DNA analysis and who gave consent for blood-based analyses and long-term follow-up.

### Variance of a quantitative trait by genotype in the presence of an interaction effect

Consider a biallelic SNP G with minor allele frequency p and in Hardy-Weinberg equilibrium. Without loss of generality and assuming an additive genetic effect of G on a quantitative trait of interest, G can be encoded as -2p, 1-2p, or 2-2p such that mean genotype equals zero. Assume that there is an additive interaction between G and a covariate C (with mean equal to zero, variance equal to one, and uncorrelated to G) such that the quantitative trait Y for the ith individual is represented as:
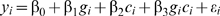
(1)Where β*_0_*, β*_1_*, β*_2_*, and β*_3_* represent constants for the intercept, the marginal effect of G, the marginal effect of C, and the interaction of G and C, respectively. It is assumed the outcome is standardized so it has unit variance conditional on G and C, that is, the error term *ε*_i_∼N(0,1). The variance in Y conditional on genotype of G can be written as:
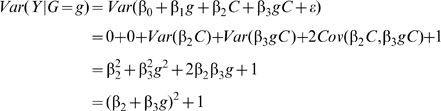
(2)Where coefficients represent true rather than estimated effects. Thus, the variance of Y will differ by genotype group (g) when β_3_≠0. Importantly, the variance does not depend on the marginal effect of G on Y (i.e. β_1_). Furthermore, as we did not specify the distribution form of the interacting covariate C, Equation 3 can be generalized to different types of interaction such as dichotomous environmental exposures, continuous environmental exposures or the three genotype classes of a biallelic SNP. The total variance of Y is given by:
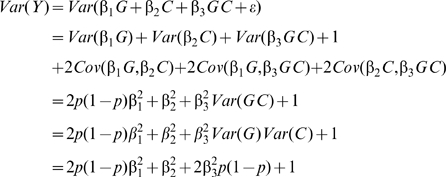
(3)Where the three covariance terms are zero because of the assumption of G and C independence. It results from equation (3) that the proportion of total variance attributable to the marginal effect of the covariate (i.e. β_2_) can be defined as:
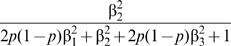
(4)Similarly, the proportion of total variance attributable to the SNP-covariate interaction (i.e. β_3_) can be defined as:
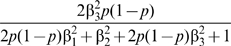
(5)


### Levene's test for equality of variance

Levene's test [12] is used to test if *k* samples have equal variances and is less sensitive to deviation from normality than the alternative Bartlett test [13]. Indeed, use of Bartlett test with empirical data led to marked inflation of type I error and was not pursued further (data not shown). The Levene test assesses the null hypothesis that the population variances are equal:

H_0_: 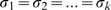

H_a_: 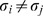 for at least one pair (*i*,*j*).

Given a variable *Y* with sample of size *N* divided into *k* subgroups, where *N_i_* is the sample size of the *i*th subgroup, the Levene test statistic is defined as:
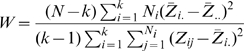
Where 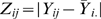 with 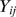 the value of Y for the *j*th observation of the *i*th subgroup, 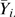 the mean of the *i*th subgroup, 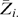 are the group means of the 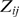 and 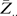 is the overall mean of the 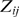.

The Levene test rejects the hypothesis that the variances are equal if:
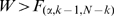
Where 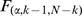 is the upper critical value of the F distribution with *k*−1 and *N*−*k* degrees of freedom at a significance level of 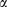.

### Simulations of interaction effects

**Figure 1:**
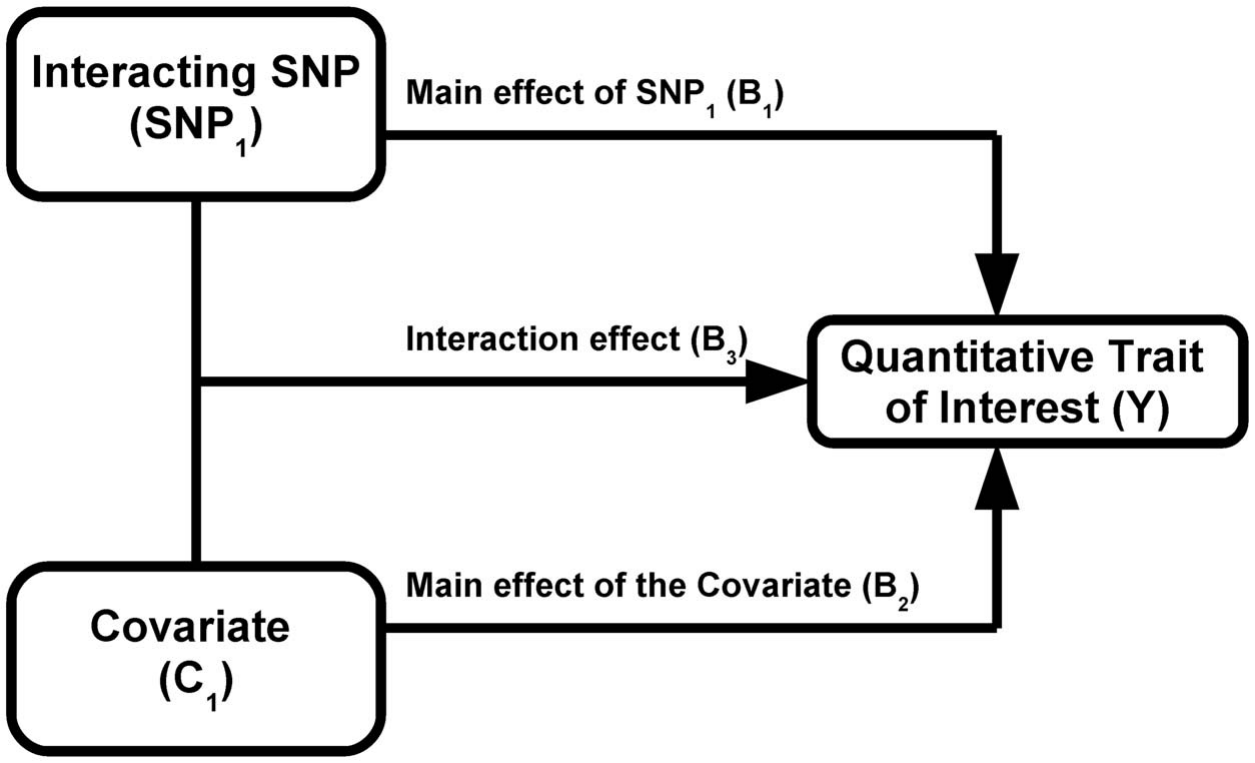
Model of interaction.

We used simulations to estimate the power of Levene's test to detect differences in variance of a quantitative trait between individuals of differing genotypes. We first simulated the case of a SNP (denoted SNP_1_) interacting with a covariate (denoted C_1_) in determining the value of a quantitative trait (denoted Y; see Figure 1). We assumed the main effect of SNP_1_ to be null in all simulations because it is expected to affect neither the within genotype variance (see Equation 2) nor the power to detect the interaction. For simplicity, only results from the additive genetic model (assuming an additive contribution of each minor allele; see Equation 1) are shown but similar conclusions can be derived from more general models of association. Under a range of models for the main effect of C_1_ (on the quantitative trait), allelic frequency of SNP_1_, and interaction between SNP_1_ and C_1_, we tested the power of Levene's test to reject the null hypothesis that the variance of Y does not vary between subsets of individuals with differing genotypes (of SNP_1_). In all simulations, the quantitative trait was assumed to have a normally distributed error term with mean equal to zero and variance equal to 1. The covariate was normally distributed with mean equal to zero and variance equal to 1. Interactions are expected to be modest at best and simulations were therefore restricted to interaction effects explaining up to 1% of the quantitative trait's total variance. Each condition was simulated 1,000 times using 15,000 individuals unless otherwise specified. In a second set of simulations, we tested the power of Levene's test of equality of variance to detect SNP-SNP interactions. These simulations are based on a model similar to the one previously introduced, with the exception that the normally distributed covariate (C_1_) is replaced by a second, independent, SNP (denoted SNP_2_). To simplify simulations, both SNPs were assigned the same allelic frequency as well as the same main effect (i.e. SNP-Y association). Varying the strength of the SNP_1_- SNP_2_ interaction, we simulated each condition 1,000 times using 15,000 individuals unless otherwise specified.

### Variance prioritization

**Figure 2:**
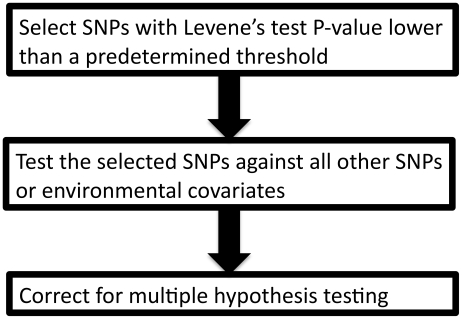
The variance prioritization procedure.

Leveraging two observations that (1) the signature of a SNP-covariate interaction may be inequality of variance between SNP genotypes and (2) a test for inequality of variance is independent of a subsequent direct test of interaction, we propose a novel method to prioritize SNPs for gene-gene and gene-environment interaction, termed “variance prioritization”. This prioritization procedure first applies Levene's test of variance equality to selects SNPs below a pre-specified threshold and then tests these “prioritized” SNPs for gene-environment or gene-gene interaction using standard linear regression models (see Figure 2), with Bonferroni correction only for the number statistical tests prioritized in the first step. Using simulation models, we explored the power of variance prioritization and compared it to an exhaustive search for interaction, without prior prioritization, followed by Bonferroni correction for multiple hypothesis testing.

Independence of variance per genotype from estimates of interaction effects under the null of no interaction is important because the validity of the method rests on having correct type I error. A proof of independence between variance per genotype and estimation of interaction effect under the null of no interaction is presented in Text S1 and Figure S1.

### WGHS participants

The study population was derived from the Women's Genome Health Study (WGHS) [14]. Briefly, participants in the WGHS include American women from the Women's Health Study (WHS) with no prior history of cardiovascular disease, cancer, or other major chronic illness who also provided a baseline blood sample at the time of study enrollment from which genomic DNA was extracted [15]. Individuals with self-reported diabetes at enrollment (or with glycated hemoglobin >7%) or taking lipid lowering medication were excluded from analysis.

All baseline blood samples underwent measurement for high-sensitivity CRP (hsCRP) via a validated immunoturbidometric method (Denka Seiken, Tokyo, Japan). CRP is an inflammatory biomarker with strong prognostic value for development of metabolic syndrome, diabetes, myocardial infarction, and stroke. Log-transformed CRP levels were used in all analyses. Soluble ICAM-1 is an inflammatory marker that has been associated with several common diseases such as diabetes, heart disease, stroke and malaria. Circulating plasma sICAM-1 concentrations were determined using a commercial ELISA assay (R&D Systems, Minneapolis, Minn.) The environmental factors tested for interaction included self-reported body mass index, menopause status, hormone replacement therapy use, smoking, age and exercise, expressed as quintiles of total energy expenditure spent from all recreational activities.

### Genotyping

DNA samples were genotyped with the Infinium II technology from Illumina. Either the HumanHap300 Duo-Plus chip or the combination of the HumanHap300 Duo and I-Select chips were used. In either case, the custom content was identical and consisted of candidate SNPs chosen without regard to allele frequency to increase coverage of genetic variation with impact on biological function including metabolism, inflammation or cardiovascular diseases. Genotyping at 318,237 HumanHap300 Duo SNPs and 45,571 custom content SNPs was attempted, for a total of 363,808 SNPs. Genetic context for all annotations are derived from human genome build 36.1 and dbSNP build 126.

SNPs with call rates <90% were excluded from further analysis. Likewise, all samples with percentage of missing genotypes higher than 2% were removed. Among retained samples, SNPs were further evaluated for deviation from Hardy-Weinberg equilibrium using an exact method and were excluded when the P-value was lower than 10^−6^. SNPs with minor allele frequency >1% in Caucasians were used for analysis. 339,596 SNPs were left after quality control. All statistical analyses were done with R, unless specified otherwise. All analyses were performed on individuals of self-reported Caucasian ancestry, with confirmation using a principal component analysis. A total of 21,799 individuals were included in our final dataset.

## Results

**Figure 3:**
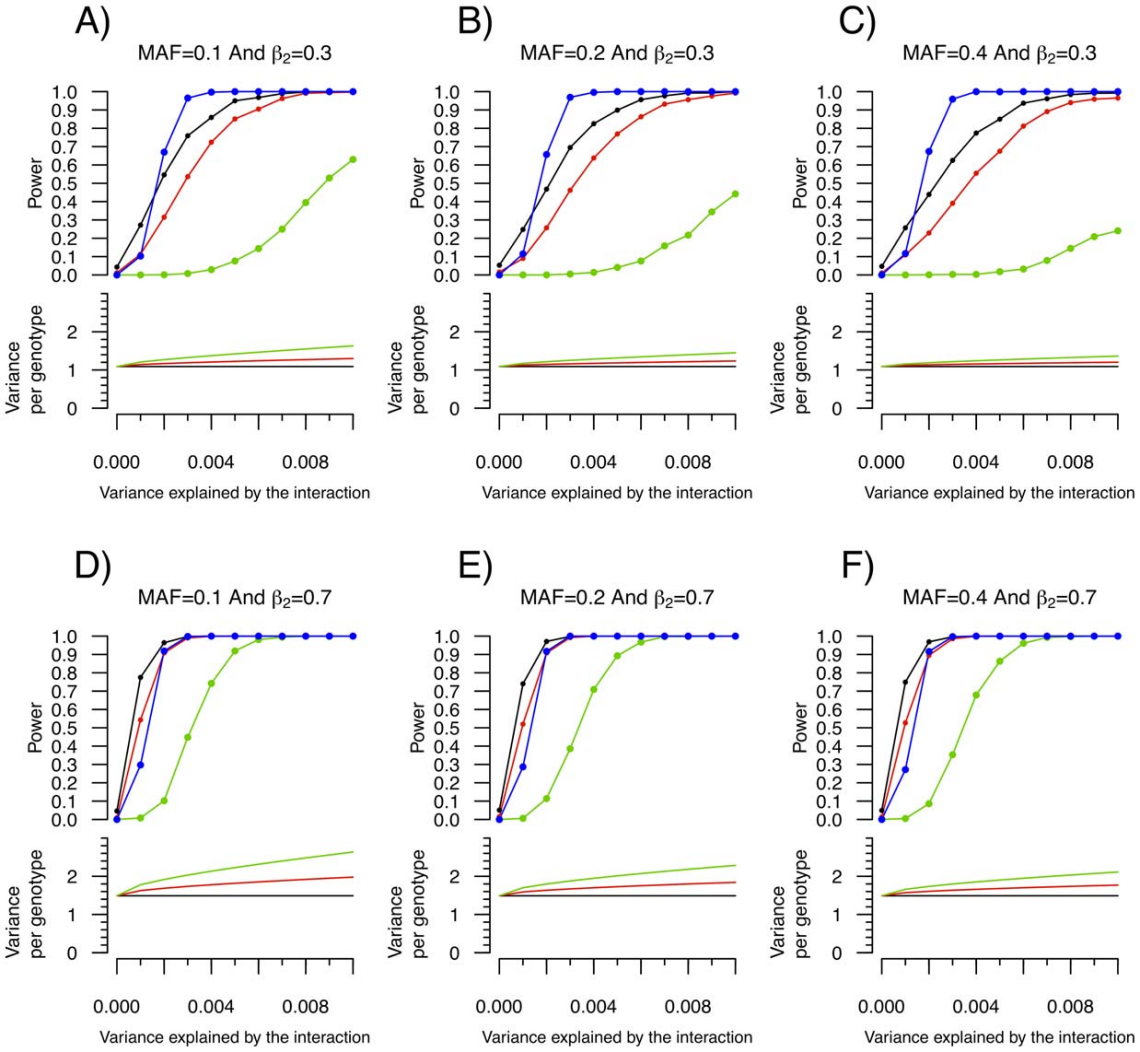
Power to detect a SNP–covariate interaction effect as function of the proportion of variance explained by the interaction. Each condition was simulated 1,000 times with 15,000 individuals according to the model shown in Figure 1. MAF refers to the minor allele frequency of SNP_1_ and β_2_ refers to the β coefficient of the C_1_-Y association. In all cases, SNP_1_ had no marginal effect (i.e. β_1_ = 0). Upper panel: Power to identify SNP_1_ as an “interacting” SNP using Levene's test with a P-value threshold of 0.05 (black), 0.01 (red) and 1.5×10^−7^ (green; to account for 340,000 tests using Bonferroni correction). Also shown is the power to detect the interaction itself with a linear regression interaction P-value cut-off of 1.5×10^−7^ (blue). Lower panel: The variance per genotype is illustrated as a function of the fraction of the total variance of the quantitative trait explained by the interaction. The homozygous major allele genotype is drawn in black, the heterozygous genotype in red and the homozygous minor allele genotype in green.

Building on the theoretical observation that the within genotype variance of a quantitative trait will vary when a genetic interaction is present, we explored the power of Levene's test of equality of variance to detect interactions. We first tested the power of Levene's test to detect a SNP (denoted SNP_1_) interacting with a single covariate, C_1_ (see Figure 1). As shown in Figure 3, the power of Levene's test to identify a SNP as interacting with a covariate was modest in most situations. For example, when the interaction effect explained 0.4% of the variance of a quantitative trait, the allelic frequency was 0.2 and the covariate was associated with the quantitative trait with β_2_ = 0.3 (explaining 8.2% of the variance; see Figure 3B), the power of Levene's test to identify a SNP as “interacting” at P<0.05 was 82.5% whereas the power of linear regression to identify the interaction at P<1.5×10^−7^ was 99.6%. In other words, even after accounting for 340,000 tests using Bonferroni correction, testing for gene-environment interactions with linear regression was more powerful than testing for inequality of variance between genotypes at nominal significance.

**Figure 4:**
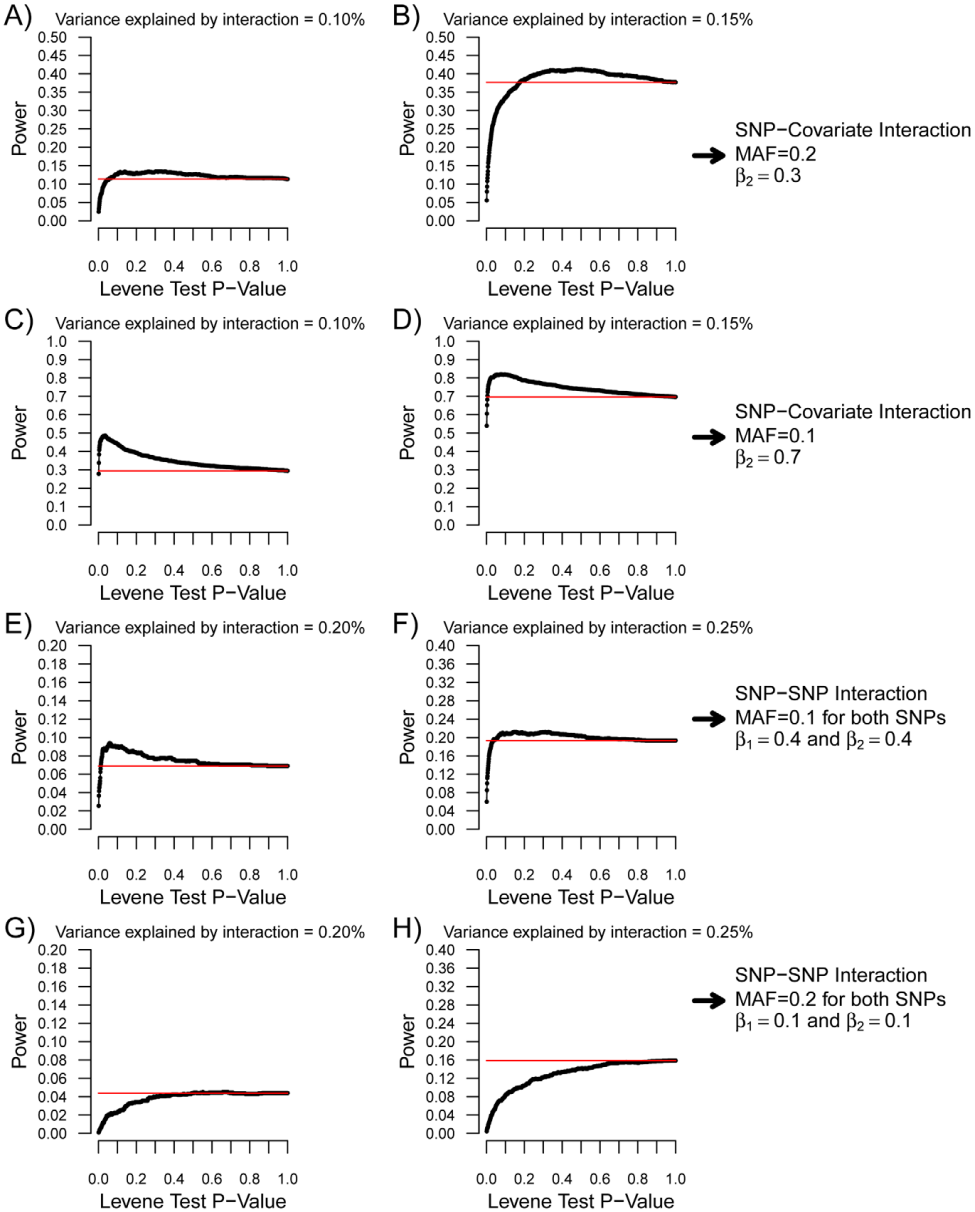
Power of variance prioritization as function of the Levene's test P-value prioritization threshold. Each condition was simulated 2,000 times with 15,000 individuals. For each condition, the Levene's test P-value threshold was varied from 0 to 1 and the power of variance prioritization calculated after accounting for multiple testing (assuming 340,000 SNPs were initially tested), as illustrated by black lines. SNP-covariate interactions were simulated in (A–D). In the case of SNP-covariate interactions, all prioritized SNPs were tested for interaction with the covariate. Red lines represent the power to detect the interaction with linear regression when correcting for all 340,000 tested SNPs (P<1.5×10^−7^). In (A,B), the minor allele frequency was set at 0.2, the covariate-Y regression coefficient (i.e. β_2_) at 0.3, and β_3_ was chosen such that the proportion of variance explained by the interaction was 0.1% and 0.15%, respectively. These conditions match those of Figure 3B. In (C,D), the minor allele frequency was set at 0.1, the covariate-Y regression coefficient (i.e. β_2_) at 0.7, and β_3_ was chosen such that the proportion of variance explained by the interaction was 0.1% and 0.15%, respectively. These conditions match those of Figure 3D. SNP–SNP interactions were simulated in (E–H). As in Figure 5, the allelic frequency of both SNPs was set to be equal, as well as the SNP-Y regression coefficient (i.e. β_1_ and β_2_). For SNP–SNP interactions, all prioritized SNPs were tested against all SNPs (not limited to prioritized ones). Red lines represent the power to detect the interaction with linear regression when correcting for all pairwise interactions between 340,000 SNPs (P<4.3×10^−13^). In (E,F), the minor allele frequency was set at 0.1, the SNP-Y regression coefficient at 0.4, and β_3_ was chosen such that the proportion of variance explained by the interaction was 0.2% and 0.25%, respectively. These conditions match those of Figure 5A. In (G,H), the minor allele frequency was set at 0.2, the SNP-Y regression coefficient at 0.1, and β_3_ was chosen such that the proportion of variance explained by the interaction was 0.2% and 0.25%, respectively. These conditions match those of Figure 5B.

Nevertheless, as can be observed in Figure 3, there were situations involving small interaction effects explaining less than 0.4% of the variance for which a nominally significant Levene's test was more powerful than linear regression testing (after multiple hypothesis correction). Because SNP effects on variance per genotype is independent of the estimates of interaction effects, variance prioritization could provide a method for decreasing the multiple testing burden in these situations, with ensuing increased power. To test the power of variance prioritization for small interaction effects, we performed further simulations in which we varied Levene's test P-value threshold used to prioritize SNPs for interaction testing (see Figure 4). For these simulations, we used the same parameters as in Figure 3B (Figure 4A and 4B) and Figure 3D (Figure 4C and 4D), fixing β _3_ such that the interaction explained 0.1% or 0.15% of total variance. We subsequently interrogated the prioritized SNPs for interaction effects using linear regression and applied a Bonferroni correction assuming 340,000 SNPs were initially tested (ex.: if Levene's test P-value cut-off is 0.01 then upon further interaction testing a SNP will be deemed significant if its interaction P-value is <0.05/(0.01×340,000)). We then compared the results of variance prioritization with a “conventional” method correcting for 340,000 tests. Each condition was simulated 2,000 times. As illustrated in Figure 4A–4D, variance prioritization was more powerful than conventional methods to detect gene-environment interactions, with the increase in power dependent on the Levene's test P-value threshold used. In almost all cases, the power was at least equal to performing an exhaustive search for gene-environment interactions with all 340,000 SNPs.

**Figure 5:**
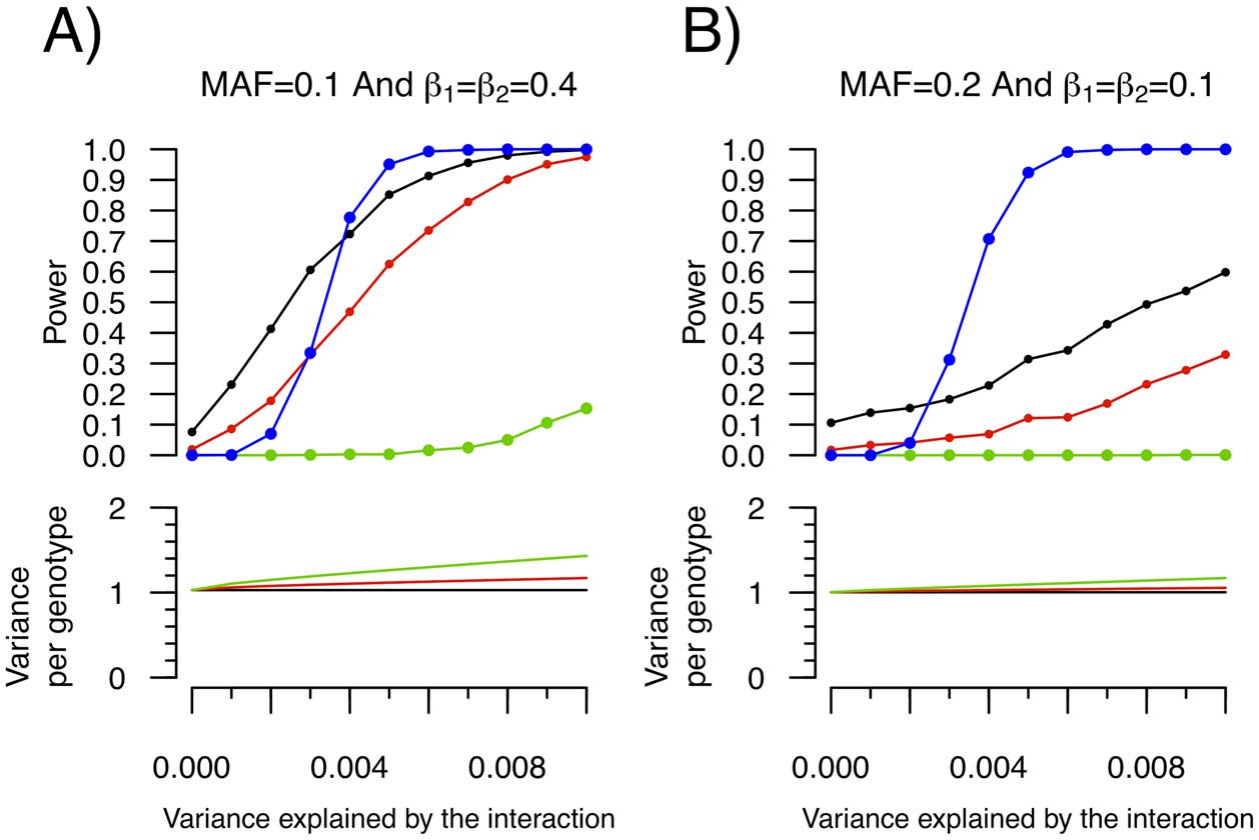
Power to detect a SNP–SNP interaction effect as function of the proportion of variance explained by the interaction. Each condition was simulated 1,000 times with 15,000 individuals. For simplicity, both SNPs were assigned the same allelic frequency (MAF) as well as the same SNP-Y β coefficient (i.e. β_1_ = β_2_). Upper panel: Power to identify either SNP_1_ or SNP_2_ as an “interacting” SNP using Levene's test with a P-value threshold of 0.05 (black), 0.01 (red) and 1.5×10^−7^ (green; to account for 340,000 tests using Bonferroni correction). Also shown is the power to detect the interaction itself with a linear regression interaction P-value cut-off of 4.3×10^−13^ (blue; chosen to account for all possible pairwise interactions between 340,000 SNPs). Lower panel: The variance per genotype is illustrated as a function of the fraction of the total variance of the quantitative trait explained by the interaction. The homozygous major allele genotype is drawn in black, the heterozygous genotype in red and the homozygous minor allele genotype in green.

Similar simulations were performed for SNP-SNP interactions (see Figure 5, and Figure 4E–4H). For simplicity, the allelic frequency of both SNPs and their main effect on the quantitative trait was set to be equal. To account for all possible pairwise SNP-SNP interactions between 340,000 SNPs, the “conventional” P-value threshold was set at 4.3×10^−13^. Figure 5 illustrates the power that either SNP will have a Levene's test P-value lower than the pre-specified cut-off, and compares it to the power to detect the interaction by exhaustive linear regression testing. Once again, the power to detect either SNP with a nominally significant Levene's test was higher than the power to identify the SNP-SNP interaction using exhaustive linear regression testing when the interaction effect was weak, providing the basis for prioritization of SNPs with Levene's test. Results of variance prioritization are shown in Figure 4E and 4F and Figure 4G and 4H, matching the conditions of Figure 5A and 5B, respectively, and fixing B_3_ such that 0.2% or 0.25% of the variance was explained by the interaction. In these simulations, all SNPs with Levene's test P-value lower than the specified threshold were prioritized and tested for interaction against all other SNPs, prioritized or not (with appropriate correction for multiple hypothesis testing). This approach was shown (by simulations) to be more powerful than testing for pairwise interactions between Levene's test prioritized SNPs only, which was underpowered as compared to an exhaustive search (data not shown). As illustrated in Figure 4E, the power of variance prioritization was superior to exhaustive search under specific conditions. For example, when a Levene's P-value lower than 0.1 was chosen as the prioritization threshold, the power of variance prioritization was 9.05% as compared to 7.30% when testing all possible pairwise interactions between 340,000 SNPs. However, variance prioritization was associated with decreased power in certain conditions, as illustrated in Figure 4G and 4H.

**Figure 6:**
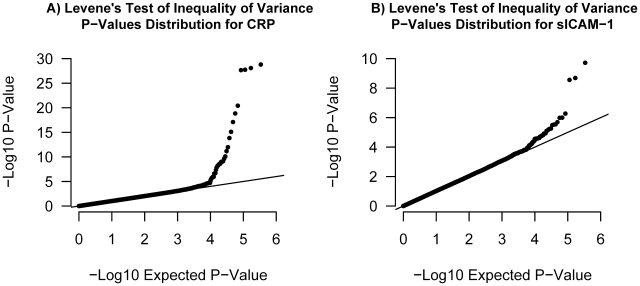
Quantile–quantile plots. Illustrated in (A) is the quantile-quantile plot of Levene's test of inequality of variance P-values applied to log-CRP for 339,596 SNPs in our set of 21,799 individuals. Illustrated in (B) is the quantile-quantile plot of Levene's test P-values applied to sICAM-1 in the same set of individuals.

To empirically test whether some SNPs exhibit inequality of variance between genotypes and whether these effects might be due to interactions, we applied Levene's test of equality of variance to ongoing genome scans of two inflammatory markers, C-reactive protein (CRP) and soluble ICAM-1 (sICAM-1). Only SNPs with genome-wide significant Levene's P-value were selected for subsequent study in order to ensure they represent true positives. From the analysis of our sample of 21,799 women, 23 SNPs had a Levene's test P-value lower than 1.5×10^−7^ for CRP (all clustering near the *LEPR* gene on chromosome 1). Because the *LEPR* SNPs are in linkage disequilibrium, only rs12753193 will be further considered since it had the most significant Levene's test P-value. Two SNPs, the *PNPLA3* SNP rs738409 and the *ICAM1* SNP rs1799969, had genome-wide significant Levene's test P-value for sICAM-1 (see Figure 6 and Table S1). To further characterize these SNPs, we then tested all pairwise interactions with the 339,595 remaining SNPs as well as environmental covariates, using linear regression. No SNP-SNP interaction was observed after correction for multiple hypothesis testing. Three SNP-environment interactions were significant after correction for multiple hypothesis testing (P<0.05/(3 SNPs×6 traits); see Table 1). The *LEPR* SNP rs12753193 interacted with BMI in the prediction of CRP levels (P = 7.2×10^−10^), the *ICAM1* SNP rs1799969 interacted with smoking status in the prediction of sICAM1 levels (P = 4.8×10^−9^) and the *PNPLA3* SNP rs738409 interacted with BMI in the prediction of sICAM1 levels (P = 1.6×10^−7^).

**Table 1:** Gene–environment interactions.

Trait	Interacting SNP	MAF	Chr	Position (Kb)	Nearest Gene	Type	Covariable	Variance of A1A1* (N)	Variance of A1A2* (N)	Variance of A2A2* (N)	Levene's P-value	Interaction P-value
CRP												
	rs12753193	0.38	1	65942.3	*LEPR*	-	BMI	1.27 (8491)	1.47 (10126)	1.68 (3167)	1.6E-29	7.2E-10
sICAM-1												
	rs1799969	0.11	19	10255.8	*ICAM1*	Missense	Smoking	6621 (17063)	5316 (4421)	4104 (300)	2.1E-09	4.8E-09
	rs738409	0.22	22	42656.1	*PNPLA3*	Missense	BMI	6087 (13098)	6743 (6965)	9205 (1110)	1.9E-10	1.6E-07

*A1A1: Homozygous Major Allele; A1A2: Heterozygous; A2A2: Homozygous Minor Allele.

We sought to determine the contribution of the three interactions to Levene's test of inequality of variance. While Levene's test P-value was 1.6×10^−29^ for the *LEPR* SNP rs12753193 in the CRP analysis, the P-value was 9.1×10^−21^ after adjustment for the interaction for BMI. Similarly, Levene's test P-values were 2.1×10^−9^ for the *ICAM1* SNP rs1799969 and 1.9×10^−10^ for the *PNPLA3* SNP rs738409 in the sICAM-1 analysis but were slightly less significant after adjustment for the intraction with BMI and smoking status, respectively, at 3.3×10^−9^ and 1.6×10^−7^. Overall, these interactions explained 0.18% of CRP total variance, and 0.16% and 0.13% of sICAM-1 total variance for the *ICAM1* and *PNPLA3* interactions, respectively. Neither the *LEPR* SNP rs12753193 nor the *PNPLA3* SNP rs738409 was associated with BMI (P>0.05). The *ICAM1* SNP rs1799969 was not associated with smoking status (P>0.05). These data collectively suggest that individual interactions are likely to have modest effects, and that additional covariates and/or SNPs might interact with the validated SNP.

## Discussion

Genetic effects on the variance of quantitative traits have been largely ignored so far in the genetic analysis. As shown in this report, differences in the variance per genotype can reflect interaction effects and these differences can be leveraged to prioritize SNPs for interaction testing. The proposed method, variance prioritization, presents the distinct advantage that it does not rely on the marginal effect of SNPs on the means to prioritize them. Importantly, it can be used in combination with prioritization based on marginal effects (on the means) if it is assumed all interacting SNPs have a main effect on the trait of interest. In such case, combining variance prioritization to prioritization based on a pre-defined marginal effect P-value provides the greatest power as compared to each procedure individually, if the assumption that interacting SNPs have main effects is true (data not shown). Another interesting property of this method is the fact that the interacting covariate need not be measured or even known to arouse suspicion of an interaction effect, perhaps leading to novel biological insight.

The goal of any genetic prioritization method is to form a subset of genetic variants enriched for the effect of interest. Variance prioritization results in substantial reduction in multiple hypothesis correction and hence increases power by using the information embedded within the variance per genotype, which we showed is independent from subsequent interaction testing. In this case, a frequentist approach was adopted since prior knowledge on the nature of the contribution of genetic interactions to complex traits is currently scarce. Our simulations have shown that the optimal Levene's test P-value threshold for variance prioritization will depend upon the allelic frequency, sample size, association of the covariate with the quantitative trait, and expected strength of the interaction. In most cases, all of these parameters can be directly observed with the exception of the interaction strength. The optimal threshold can therefore be empirically selected through simulations when the potentially interacting covariate (or SNP) is characterized and the expected interaction effect size defined.

Inequality of variance between genotypes could be the result of factors other than interactions, such as increased variability. Furthermore, the heteroscedacity of variances also depends on an assumption of homogeneity of variances within genotype, which could be violated if variances tend to be proportional to means. This might reflect an inappropriate choice of scale for the phenotype and a variance-stabilizing transform like the log or square root might then be indicated. Linkage disequilibrium with variants of large effect sizes could also theoretically lead to inequality of variance between genotypes. Conversely, non-additive interactions or SNPs with a multiplicity of interactions may not result in deviation of Levene's test. Failure to prioritize “interacting” SNPs or prioritization of “non-interacting” SNPs can theoretically lead to decreased statistical power but should not lead to inflated type I error since the first step is a screening test followed by confirmatory analysis. While care was taken to simulate models with plausible effect sizes (i.e. explaining less than 1% of total variance), the scenarios described in this report undoubtedly represent simplified situations. Nevertheless, these simulations suggest that variance prioritization has increased power to detect genetic interactions of very modest strength in situations where conventional methods perform poorly.

Genetic effects on variance in an empirical dataset led to the identification of three novel interactions. Given the strength of the observed interactions, they would have also been identified with traditional methods. The significance of these results resides in (1) the existence of SNPs with inequality of variance between genotypes at genome-wide significance and (2) the presence of biologically plausible interactions between these SNPs and environmental factors. The first interaction involved the leptin receptor SNP rs12753193 in the prediction of CRP. Leptin is a protein synthesized and secreted by the adipose tissue whose main function is to regulate appetite trough interaction with the leptin receptor in the hypothalamus [16]. As such, leptin levels are directly proportional to BMI and patients deficient in either leptin or leptin receptor are characterized by marked obesity [17]. Interestingly, *LEPR* is also expressed by the liver, the source of circulating plasma CRP, and physiological concentrations of leptin stimulate expression of CRP in primary hepatocytes [18]. This might explain why only a small fraction of the inequality of variance between the *LEPR* SNP rs12753193 genotypes was due to the interaction with BMI: the statistical interaction may only capture a small fraction of the underlying biological interaction between leptin levels and its receptor.

The *LEPR* SNP rs12753193 is known to be associated with CRP concentration at a genome-wide level [19]. The minor allele of rs12753193 (i.e. “G”) is associated with lower levels of CRP and was shown in this report to be associated with a greater effect of BMI on CRP concentrations. This confirms the soundness of using marginal effects of SNPs to select a subset for interaction testing. No other SNP with genome-wide significance for CRP showed evidence of inequality of variance between genotypes (excluding SNPs in linkage disequilibrium with rs12753193).

The second interaction observed involved the *ICAM1* SNP rs1799969 in the prediction of sICAM-1 levels. The latter SNP is a coding non-synonymous SNP (G241R) known to be associated with sICAM-1 levels [20]–[22] and was shown in this report to interact with smoking status. Smoking is a very strong determinant of sICAM-1 levels (explaining up to 13% of sICAM-1 total variance) and was associated with a stronger effect of rs1799969 in WGHS. Interestingly, an interaction between another *ICAM1* non-synonymous SNP (rs5498, r^2^ = 0.17 with rs1799969) and smoking was recently described with regards to susceptibility to coronary artery disease [23].

The third interaction identified involved the *PNPLA3* SNP rs738409 in the prediction of sICAM-1. *PNPLA3* encodes a protein of unknown function that belongs to the patatin-like phospholipase family. The methionine allele of the missense *PNPLA3* SNP rs738409 (Ile148Met) has recently been associated with increased hepatic fat levels, hepatic inflammation and plasma levels of liver enzymes (traits often linked to insulin resistance and obesity) [24]. Interestingly, that same variant has been associated with insulin secretion in response to oral glucose tolerance test [25]. Levels of the inflammatory marker sICAM-1 are known to be correlated with insulin resistance and obesity [26]. Consistent with this literature, the methionine allele was associated with sICAM-1 itself (P = 2.2×10^−7^; Beta = 4.9 ng/mL per minor allele) while being associated with a greater effect of BMI on sICAM-1 levels.

In this report, we demonstrated that the presence of gene-gene or gene-environment interactions can result in changes in variance of a quantitative trait per genotype and that these changes can be exploited to prioritize SNPs for interaction testing. We used this property of quantitative trait interactions on a real dataset and discovered three new, biologically relevant, interactions. Variance prioritization presents two advantages over other methods. First, it does not depend on the marginal effect of SNPs on the means. Second, it can identify SNPs likely to be involved in an interaction even when the interacting genetic or environmental covariate(s) is not measured. For instance, had BMI or smoking status not been assessed in the WGHS, the *LEPR* SNP rs12753193, the *ICAM1* SNP rs1799969 and the *PNPLA3* SNP rs738409 would still have been selected as candidates for interaction effects, perhaps leading to novel biological hypotheses.

## Supporting Information

Figure S1 (A) QQ-plot of interaction P values in the presence of inequality of variance between genotypes (95% CI). (B) QQ-plot of interaction P values when selecting SNPs with Leneve's P<0.05 (95% CI).
(0.61 MB TIF)

Table S1 SNPs with Levene's test of inequality of variance P-value lower than 1.5×10^−7^ from the analysis of C-reactive protein and soluble ICAM-1 in 21,799 women from the WGHS.
(0.07 MB DOC)

Text S1 Proof of independence between variance per genotype and estimation of interaction effect under the null of no interaction.
(0.07 MB DOC)
