# Supplementary figures and images for: On the Use of Variance per Genotype as a Tool to Identify Quantitative Trait Interaction Effects: A Report from the Women's Genome Health Study
Source: PLoS Genet. 2010 Jun 17;6(6):e1000981. doi: 10.1371/journal.pgen.1000981 (PMC2887471; doi:10.1371/journal.pgen.1000981)

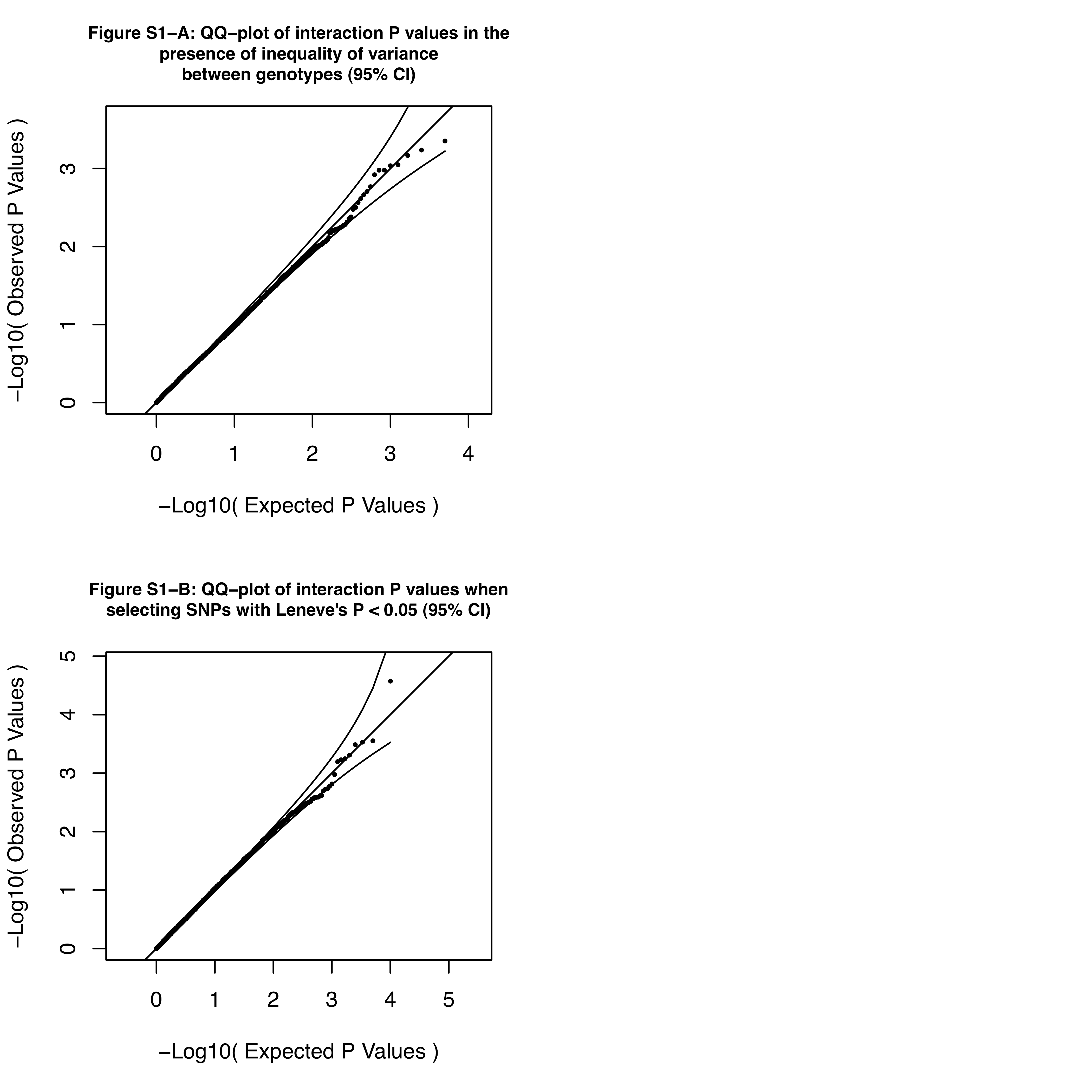

Supplement: Figure S1 — (A) QQ-plot of interaction P values in the presence of inequality of variance between genotypes (95% CI). (B) QQ-plot of interaction P values when selecting SNPs with Leneve's P<0.05 (95% CI). (0.61 MB TIF) [file pgen.1000981.s001.tif]
