# Supplementary material for: On the Use of Variance per Genotype as a Tool to Identify Quantitative Trait Interaction Effects: A Report from the Women's Genome Health Study
Source: PLoS Genet. 2010 Jun 17;6(6):e1000981. doi: 10.1371/journal.pgen.1000981 (PMC2887471; doi:10.1371/journal.pgen.1000981)
Supplement: Table S1 — SNPs with Levene's test of inequality of variance P-value lower than 1.5×10^−7^ from the analysis of C-reactive protein and soluble ICAM-1 in 21,799 women from the WGHS. (0.07 MB DOC) [file pgen.1000981.s002.doc]

**Table S1: SNPs with Levene’s test of inequality of variance P-value lower than 1.5 x 10^-7^** from the analysis of C-reactive protein and soluble ICAM-1 in 21,799 women from the WGHS

| **Trait** | **SNP** | **Levene's Test P-Value** | **Chr.** | **Position (Kb)** | **MAF** | **Function** | **Nearest Gene** | **Marginal Effect**  **P-value** | **Marginal Effect Rank** |
| --- | --- | --- | --- | --- | --- | --- | --- | --- | --- |
| LNCRP | rs6657868 | 6.8E-12 | 1 | 65686.3 | 0.38 | intron | LEPR | 3.1E-15 | 52 |
| LNCRP | rs6588147 | 2.3E-10 | 1 | 65708.1 | 0.34 | intron | LEPR | 8.4E-14 | 59 |
| LNCRP | rs12409877 | 1.1E-12 | 1 | 65716.5 | 0.40 | intron | LEPR | 1.5E-15 | 49 |
| LNCRP | rs1782754 | 9.5E-10 | 1 | 65765.9 | 0.27 | intron | LEPR | 2.0E-14 | 56 |
| LNCRP | rs1171269 | 1.0E-09 | 1 | 65769.4 | 0.27 | intron | LEPR | 2.1E-14 | 57 |
| LNCRP | rs1022981 | 7.5E-10 | 1 | 65772.6 | 0.27 | intron | LEPR | 1.7E-14 | 55 |
| LNCRP | rs1137100 | 3.7E-09 | 1 | 65809 | 0.27 | Missense | LEPR | 2.1E-13 | 61 |
| LNCRP | rs4655537 | 1.4E-19 | 1 | 65831.4 | 0.36 | intron | LEPR | 4.8E-21 | 38 |
| LNCRP | rs6665672 | 4.3E-08 | 1 | 65841.6 | 0.17 | intron | LEPR | 5.2E-09 | 80 |
| LNCRP | rs8179183 | 5.7E-09 | 1 | 65848.5 | 0.17 | Missense | LEPR | 5.1E-10 | 73 |
| LNCRP | rs3790437 | 8.3E-09 | 1 | 65858 | 0.17 | intron | LEPR | 7.3E-10 | 74 |
| LNCRP | rs1892534 | 1.8E-28 | 1 | 65878.5 | 0.39 | - | LEPR | 7.4E-42 | 19 |
| LNCRP | rs2186245 | 1.5E-14 | 1 | 65890.7 | 0.19 | - | LEPR | 1.5E-23 | 33 |
| LNCRP | rs12022410 | 3.8E-21 | 1 | 65926.5 | 0.45 | - | LEPR | 8.4E-26 | 32 |
| LNCRP | rs2211651 | 2.2E-28 | 1 | 65928.6 | 0.38 | - | LEPR | 7.7E-42 | 20 |
| LNCRP | rs2889195 | 8.3E-29 | 1 | 65929.3 | 0.38 | - | LEPR | 4.2E-42 | 18 |
| LNCRP | rs12753193 | 1.6E-29 | 1 | 65942.3 | 0.38 | - | LEPR | 3.2E-42 | 17 |
| LNCRP | rs7539471 | 8.0E-18 | 1 | 65958 | 0.31 | - | PDE4B | 8.0E-14 | 58 |
| LNCRP | rs4291477 | 7.9E-16 | 1 | 65963.7 | 0.46 | - | PDE4B | 9.7E-19 | 41 |
| LNCRP | rs5010905 | 2.4E-09 | 1 | 65971.8 | 0.26 | - | PDE4B | 3.5E-15 | 53 |
| LNCRP | rs12131840 | 1.4E-08 | 1 | 66004.4 | 0.14 | - | PDE4B | 4.3E-12 | 65 |
| LNCRP | rs12739892 | 8.0E-11 | 1 | 66077.2 | 0.23 | intron | PDE4B | 7.9E-08 | 95 |
| ICAM | rs1799969 | 2.1E-09 | 19 | 10255.8 | 0.11 | Missense | ICAM1 | 1.4E-87 | 1 |
| ICAM | rs738409 | 1.9E-10 | 22 | 42656.1 | 0.22 | Missense | PNPLA3 | 2.2E-07 | 61 |
