# Supplementary material for: On the Use of Variance per Genotype as a Tool to Identify Quantitative Trait Interaction Effects: A Report from the Women's Genome Health Study
Source: PLoS Genet. 2010 Jun 17;6(6):e1000981. doi: 10.1371/journal.pgen.1000981 (PMC2887471; doi:10.1371/journal.pgen.1000981)
Supplement: Text S1 — Proof of independence between variance per genotype and estimation of interaction effect under the null of no interaction. (0.07 MB DOC) [file pgen.1000981.s003.doc]

**Proof of independence between variance per genotype and estimation of interaction effect under the null of no interaction**

Independence of variance per genotype from estimates of interaction effects under the null of no interaction is important because the validity of the method rests on having correct type I error. The aims of this proof are to demonstrate (1) that there is independence of variance per genotype and estimates of interaction effects under the null of no interaction, and (2) that even when variance varies between genotypes for reasons other than interaction, then independence will be maintained. When an interaction effect is present, then the variance per genotype will be correlated to the interaction effect. This proof assumes a simple model of interaction (involving two genotypes although it can be extended to more than two as shown later). In keeping with the reminder of the manuscript, more complex models of interactions are not considered here. There are specific situations where variance per genotype may not vary in the presence of an interaction, but we limit ourselves to the very general linear models presented throughout.

Assume the following model for an interaction effect according to genotype:

In this model, y_1_ is a vector representing the values of a quantitative trait for a given genotype while y_2_ represents values for individuals with a different genotype (taking for granted there are only two possible genotypes). Also, X represents the observed values of a covariate and the σ’s are the within genotype standard deviations. According to this model, the variance per genotype is given by Var(y_1_) = β_1_^2^ Var(X_1_) + σ_1_^2^ and Var(y_2_) = β_2_^2^ Var(X_2_) + σ_2_^2^. Hence, assuming σ_1_^2^ = σ_2_^2^, variance per genotype will be equal if β_1_  = β_2_ but will be unequal if the effect of X on y varies according to genotype (i.e. β_1_  ≠ β_2_), that is, if there is a genetic interaction effect. The maximum likelihood estimator of β_i_ is given by:

And the maximum likelihood estimator of σ_i_^2^ is (N_i_ being the number of individuals with genotype “i”):

Using the substitution y_i_=X_i_β_i_ + ε_1_ where ε_1_~N(0, I_i_σ_i_^2^) and β_i_ is the true value of the coefficient, it follows that:

And:

So that:

Similarly:

It then follows that:

And

Estimates of the interaction effect are therefore independent from within genotype standard deviations (σ_i_) and thus from the variance per genotype under the null of no interaction.. Further, because we did not assume equal variance per genotype (i.e. σ_1_¹σ_2_), it follows that estimates of the interaction effect will not be biased even when there is a true difference in the variance per genotype (for reasons other than an interaction effect). This proof can be extended to cases where more than two genotypes are possible since will be equal to 0 for any pairs of genotypes a¹b and c¹d.

We also tested for type I error inflation using simulated data. In a first set of simulations, a SNP was randomly generated and a quantitative trait subsequently simulated such that the mean was equal for every genotype but the variance per genotype varied. Briefly, we simulated 15,000 individuals assuming an allelic frequency of 0.4 and standard deviations of 1.0, 1.1 and 1.2 for genotypes with 0, 1 and 2 minor alleles, respectively. We then tested for interaction with a simulated covariate and noted no inflation of type I error after 5,000 simulations (see Figure S1-A), despite the significant effect on variance per genotype (median Levene’s P-value of 2.9 x 10^-24^). In a second set of simulations, we randomly generated a SNP and a quantitative trait independently of each other. We then selected SNPs with significant Levene’s test P-value (P<0.05) and tested them for interaction effects with a simulated covariate. Briefly, we again simulated 15,000 individuals assuming an allelic frequency of 0.4. After 200,0000 simulations, 10,060 SNPs had a Levene’s P-value < 0.05. No inflation of type I error was noted when these 10,060 SNPs were tested for interaction (Figure S1-B). In other words, even when the within genotype variance varies (for any other reason than a genetic interaction), the type I error of subsequent interaction testing is well controlled.
